# The Buzz on Insecticides: A Review of Uses, Molecular Structures, Targets, Adverse Effects, and Alternatives

**DOI:** 10.3390/molecules28083641

**Published:** 2023-04-21

**Authors:** Maria F. Araújo, Elisabete M. S. Castanheira, Sérgio F. Sousa

**Affiliations:** 1 UCIBIO/REQUIMTE, BioSIM—Department of Medicine, Faculty of Medicine, University of Porto, Alameda Prof. Hernâni Monteiro, 4200-319 Porto, Portugal; 2 Associate Laboratory i4HB—Institute for Health and Bioeconomy, Faculty of Medicine, University of Porto, 4200-319 Porto, Portugal; 3 Physics Centre of Minho and Porto Universities (CF-UM-UP), University of Minho, Campus de Gualtar, 4710-057 Braga, Portugal; 4 Associate Laboratory LaPMET, University of Minho, Campus de Gualtar, 4710-057 Braga, Portugal

**Keywords:** insecticides, pest management, environmental sustainability, carbamates, organophosphates, neonicotinoids, organochlorines, biological control, resistance, biopesticides

## Abstract

Insecticides play a critical role in controlling the spread of insect-borne diseases and preserving crop health. These chemical substances are specifically formulated to kill or manage insect populations. Over the years, various types of insecticides have been developed, including organophosphates, carbamates, pyrethroids, and neonicotinoids, each with unique modes of action, physiological targets, and efficacy. Despite the advantages that insecticides offer, it is imperative to recognize the potential consequences on non-target species, the environment, and human health. It is therefore crucial to follow recommended label instructions and employ integrated pest management practices for the judicious use of insecticides. This review article provides an in-depth examination of the various types of insecticides, including their modes of action, physiological targets, environmental and human health impacts, and alternatives. The aim is to furnish a comprehensive overview of insecticides and to emphasize the significance of responsible and sustainable utilization.

## 1. Introduction

The definition of insecticide is any toxic substance that is used to eradicate and control insect populations (these include ovicides and larvicides for eggs and larvae, respectively). Such compounds are primarily used to control pests that infest cultivated plants, or to eliminate disease-carrying insects in specific areas. The earliest documented insecticide compounds were substances such as sulfur, heavy metals, salts, and even plant extracts (e.g., *Chrysanthemum cinerariifolium* formerly known as *Dalmatian pyrethrum*) [1,2,3,4]. The use of elemental and/or natural compounds for pest control started at the very dawn of agriculture and has continued, in some cases, to be used to the present day. The first record of insecticide usage dates ≈4500 years ago by Sumerian people, who used sulfur compounds in order to kill insects and mites. Additionally, ≈3200 years ago, the Chinese were using mercury and arsenical compounds to control body lice [5]. Botanical preparations are also amongst the first recorded pest controllers. For instance, the discovery of *C. cinerariifolium* insecticidal activity may have been accidental. A book about these common flowers tells us the story of a German woman of Dubrovnik who picked the flowers for their beauty, and after they withered, she noticed that dead insects had gathered around the plant’s remnants, suggesting a possible connection between *C. cinerariifolium* and its ability to kill insects [4]. These flowers, formerly classified as *pyrethrum* flowers, contain up to 1.5% of a substance named pyrethrin, which is an active insecticidal compound [3]. This ingredient was used as an insecticide in ancient China and in the Middle Ages in Persia, and it was brought to Europe shortly after by Armenian traders, being sold as “Persian dust” (around ≈200 years ago). This powder was produced from dried flowers of *Chrysanthemum roseum*, and the major constituents of these dried extracts were pyrethrin I and II, which compose some of today’s household sprays [6].

In the 19th century, a vast variety of chemicals started to be used against crops’ infestations. A farmer discovered that *Paris green*, a paint pigment (copper acetoarsenite), had supposedly insecticidal properties when discarding remaining paint onto a potato plantation that was infested with the CPB (Colorado potato beetle) [7]. This substance was widely used in many countries of the world until the mid-20th century. In order to control the malaria vector, *Paris green* would be sprayed on the surface of breeding places, working as a larvicide [8]. Around the same time period, borax was also reported as an insecticide when used as a coating material for crop seeds such as corn [9]. 

During the late 1800s and early 1900s, scientists developed the first synthetic organic chemicals that served as insecticides. These modern synthetic insecticides were made in the form of organochloride compounds. Although benzene hexachloride (BHC) and dichlorodiphenyltrichloroethane (DDT) were synthesized in the 1800 s, it was not until later that their insecticidal properties were fully discovered and utilized [10]. Michael Faraday, an English scientist, first produced BHC in 1825, while Othmar Ziedler, an Austrian chemist, synthesized DDT in the same year. However, it was not until Bender and Müller, respectively, in 1933 and 1939, that the insecticidal properties of BHC and DDT were first demonstrated [11]. This was probably the most significant development in the history of pest control and resulted in Müller being awarded the Nobel Prize in 1948 [12]. This chemical agent was designed to eliminate insects, weed, rodents, fungi, and other human annoyance trouble, but its adverse effects spread to every ecosystem it came into contact with. In fact, it still impacts the environment and human health to the present day, due to its long residual efficacy and accumulation throughout the food chain [13]. A milestone in environmental science is the publication of the book *Silent Spring* by Rachel Carson [14] that exposes the effects of the indiscriminate use of pesticides such as DDT; this book was considered one of the greatest science books of all time. Regarding human health, DDT is the cause of various ailments, including various types of cancer, acute and persistent injuries to the nervous system, lung damage, injury to reproductive organs, and dysfunction of the immune and endocrine systems, and it has also been linked to numerous birth defects [13]. DDT quickly lost its popularity as the USA, Japan, and Western Europe banned the production and application of the substance (in the Stockholm Convention, 2001), classifying it as a priority pollutant. However, it is continuing to be illegally used in third-world countries [15].

Shortly thereafter, at the beginning of the 20th century, researchers began exploring modifications to natural pyrethrins’ structure. In 1949, Schechter and LaForge discovered allethrin, the first pyrethroid compound, which improved the effectiveness of insecticides over time [16]. These compounds were divided into two types, Type I and Type II, based on their chemical structure. The discovery of allethrin, which belongs to the Type I pyrethroid compound group, renewed interest in pyrethrins as insecticides [17]. It also inspired chemists worldwide to investigate modifications to the pyrethroid alcohol and acid moieties, and eventually to the essential ester function [16]. These derivatives proved to be significantly more effective, cost-effective, and stable than their natural pyrethrin counterparts [18]. Despite the fact these synthetic compounds lose their activity rather quickly when exposed to ultraviolet light, this photodegradation property of pyrethroids [19] helped to prevent their accumulation in the environment, and, therefore, this class of insecticides still finds wide application in plant protection.

In more recent years, we have seen the appearance of new insecticides, such as neonicotinoids, a class of neuro-active insecticides that are chemically similar to nicotine; they act by systematically moving in the plant tissues and protecting all parts of the plant. Reportedly, their discovery is connected to the Shell and Bayer companies, which started their development in the 1980s and 1990s, respectively [20]. However, imidacloprid was the very first neonicotinoid that appeared on the insecticide market. It was registered as “Hachikusan” in Japan in 1993 [3]. Nowadays, it is possible to find a large number of represented neonicotinoids, such as acetamiprid, clothianidin, dinotefuran, imidacloprid, nitenpyram, nithiazine, thiacloprid, and thiamethoxam [21]. They quickly gained popularity, and neonicotinoids such as imidacloprid have been the most widely used insecticides in the world, from 1999 to at least 2018 [22]. In 2016, imidacloprid was banned alongside clothianidin, thiamethoxam, acetamiprid, and thiacloprid by the French government, and the EFSA (The European Food Safety Authority) concluded, in February 2018, that the most used neonicotinoid insecticides represent a risk to wild bees and honeybees [23].

Currently, besides neonicotinoids (especially imidacloprid), the other two most used insecticides are organophosphates (more specifically chlorpyrifos) and carbamates (more specifically carbaryl). Organophosphate insecticides correspond to roughly half of all insecticides used worldwide, with chlorpyrifos being the most widely used (approved to be used on more than 50 different crops) [24]. Regarding carbamate insecticides, there are ≈50 chemicals that belong to this family and are used as fungicides, herbicides, and nematicides, in addition to being used as insecticides [25,26]. Carbaryl, a white crystalline solid, was the first carbamate to be commercialized, and to this day, it is more widely used than all the other carbamates combined [24].

## 2. Insecticides: Importance and Increasing Demand

From ancient times to the present day, the use of pesticides such as insecticides has become an essential and strictly necessary agricultural component in order to assure crop yields and minimize post-harvest losses [27]. With a continuously increasing population, in addition to deteriorating environmental conditions (based on irrefutable and growing evidence of climate change coupled to increasing levels of pollution), the task of achieving long-term development without causing environmental harm has never been greater. In a world where aliment production must grow by 70 to 100% by 2050, in order to meet the food demand of a population of more than 9 billion people, agriculture is one of the major challenges for sustainable development [28]. With agriculture being the primary cause of deforestation [29], already occupying 70% of the world’s grasslands, 50% of savannas, and 45% of temperate forests [28], there is an increasing need to limit crops terrain usage, while also increasing yields to sustain demand. Additionally, the changing of dietary habits of expanding middle classes has driven the need for higher quality products through the control of various insect pests [30]. Furthermore, insects are often hosts of devastating diseases. Vector-borne diseases are among the major causes of illness and death worldwide, particularly in tropical and subtropical regions; therefore, vector control, through the use of insecticides, is highly important for the prevention and control of infectious diseases such as malaria, dengue, and filariasis [31]. Available approaches to control pest insects range from (natural or chemical) insecticide usage to cultural practices (e.g., crop rotation), genetically modified plants (e.g., increasing host plant resistance), biological control (e.g., the release of sterilized pests to disrupt reproduction), physical and mechanical control, and microbial control [32]. Sometimes, multiple approaches are needed in order to address certain infestation problems; however, for many pest control complications, insecticides have and continue to provide farmers and public health workers with the tools and means to predictably, quickly, and effectively address a specific pest problem [30]. Insecticides are often an easy and reliable solution, which results in an increasing demand for the compounds. Nonetheless, their toxicology should be thoroughly studied before being applied in order to prevent any more environmental residual and prolonged damage.

## 3. Prominent Insecticides and Their Adverse Effects

Environmental contamination is the main problem associated with these poisonous compounds, and they may be harmful to other organisms, including humans, rather than just exclusively killing insects. Many insecticides are short-lived and decompose quickly or are fully metabolized by the animals that ingest them, but some are persistent and, when administrated in higher quantities, could be devastating for ecosystems, as they travel across the food chain. When insecticides are applied to crops, much of it reaches the soil and consequently contaminates groundwater reserves from direct application or, in worst case scenarios, as runoff from treated areas. Furthermore, when poorly used, insecticides could create some levels of resistance amongst an insect population. They could also eliminate the natural predators that once held them back. The nonspecific nature of the currently used broad spectrum of chemicals makes them more likely to have such unintended effects on the abundance of both harmful and beneficial insects. In the following table (Table 1), the top ten most used insecticides in the world at the moment are listed. This table includes their structural and chemical description. Additionally, their adverse effects are also indicated.

## 4. Major Known Targets for Insecticidal Activity

To fully comprehend how an insecticide works, it is necessary to have knowledge about its particular target(s) within an organism. Typically, this is a crucial protein or enzyme. Consequently, insecticides are usually classified based on their structure and mode of action. Most insecticides act on (1) the insect’s nervous systems, (2) metabolic targets, and (3) growth regulators and others. 

### 4.1. Molecules Disrupting Insect’s Nervous Systems

The primary target observed for most insecticides is the peripheral nervous system (PNS) and central nervous system (CNS). 

Organochlorines are a type of insecticide that is made up of organic compounds that contain one or more covalently bonded chlorine atoms. The most well-known type of organochlorine is chlorinated hydrocarbons, which include DDT, chlordane, lindane, and endosulfan [50]. Through an imbalance of sodium and potassium ions, these insecticides disrupt nerve impulse transmission. Furthermore, some organochlorines act on GABA receptors, preventing ions from entering neurons and resulting in a hyperexcitable state characterized by tremors and convulsions [51].

Other insecticides that target GABA receptors include antibiotic insecticides and pyrethroids. 

Antibiotic insecticides, also known as microbial insecticides, are derived from bacteria or fungi and are effective against tough greenhouse pests such as spider mites and leaf miners. These insecticides block neurotransmitters at the neuromuscular junction, impairing the insects’ ability to feed and lay eggs and ultimately leading to their death [52,53,54]. Spinosyns are a very special type of microbial insecticides with a complex structure that includes a large macrocyclic lactone ring, a tetra-hydrogen ring, and a dihydropyranone group, as well as oxygen and nitrogen atoms and a sugar moiety [55]. These compounds are extremely specific and can effectively target a wide variety of pests, including caterpillars, lepidopteran larvae, leaf miners, thrips, and termites [56].

On the other hand, pyrethroids (Table 1, molecules 8–10) are made up of a cyclopropane or cyclohexane ring with a carboxylic acid group attached to it and two aryl or heteroaryl groups that may contain halogen or other substituents [57]. This structure enables the molecule to bind to the sodium channel in the nerve cell membrane of insects, causing a sodium/potassium imbalance that causes the insect to become hyperexcitable. Tremors, incoordination, hyperactivity, and, finally, paralysis are symptoms [58]. Pyrethroids are typically classified into two types: type I and type II. Type I pyrethroids have an alfa-cyano group attached to the cyclopropane or cyclohexane ring, whereas type II pyrethroids have a beta-cyano group attached to the same ring, making them more potent and persisting in the environment for longer [57]. They, like others, are extremely toxic to fish, in addition to being effective against most agricultural pests [58].

Both neonicotinoids and formamidines are new classes of insecticides that are applied at low dosages and are extremely effective.

Neonicotinoids (Table 1, molecules 1 and 6), named for their chemical similarity to nicotine, consist of a heterocyclic ring structure to which a nitro group and a cyan group are attached. Neonicotinoids act as an activator of nicotinic acetylcholine receptors (nAChRs) in the nervous system of insects, leading to overstimulation and paralysis. Their high affinity for insect nAChRs and longer half-life compared to nicotine make them highly effective insecticides. One of the most significant advantages of neonicotinoids is their high selectivity in toxicity, meaning that they have a minimal impact on non-target organisms such as birds and mammals. However, their use has been the subject of controversy due to their potential impact on pollinators such as bees [59].

Formamidines are a type of pesticide that works by blocking the monoamine oxidase enzyme, which is responsible for the breakdown of neurotransmitters in insects, and formamidines create a buildup of these molecules by blocking it. This causes infected insects to become dormant, eventually leading to death. Some of the most often used formamidine compounds in pesticides are chlordimeform, amitraz, and thiacloprid. Formamidines have various advantages over conventional insecticides, including low mammalian toxicity and the fact that they do not last long in the environment. These chemicals are commonly used to manage pests that have evolved resistance to other pesticide classes, such as organophosphates and carbamates [60].

Organophosphates (Table 1, molecules 2, 4, 5, and 7) and carbamates (Table 1, molecule 3) both block AChE and produce acetylcholine buildup at NMJs, resulting in the fast twitching of voluntary muscles and paralysis [61]. They share a similar chemical structure, but with some key differences—both contain a central phosphorus or carbamate functional group, respectively, that is important for their insecticidal properties. 

Organophosphates contain a phosphorus atom that is typically bonded to two oxygen atoms (forming a carbonyl group). This carbon-oxygen double bond is essential for organophosphates’ ability to inhibit AChE activity. Malathion, chlorpyrifos, and parathion are examples of organophosphate insecticides [61].

Carbamates, on the other hand, have a carbamate functional group consisting of a nitrogen atom bonded to both a carbonyl group and an oxygen atom. Like organophosphates, carbamates inhibit the activity of AChE but in a slightly different way—instead of forming a covalent bond with the enzyme like organophosphates do, carbamates form a reversible bond with AChE. Examples of carbamates include carbaryl, methyl and aldicarb [62].

Botanical preparations remain a popular choice as insecticides, with some even serving as the basis for synthetic insecticides such as pyrethroids (derived from pyrethrum) and neonicotinoids (inspired by the use of tobacco for crop protection). One such compound is limonene, a terpene found in the essential oils of citrus fruits, rosemary, and peppermint. Limonene targets the sensory nerves of the peripheral nervous system and can effectively control fleas, lice, mites, and ticks. Furthermore, it has low toxicity to warm-blooded animals and only minor toxicity to fish. However, botanical preparations may require more frequent application than synthetic insecticides. Despite this, they can be a viable option for integrated pest management programs that prioritize non-chemical control methods.

**Figure 1:**
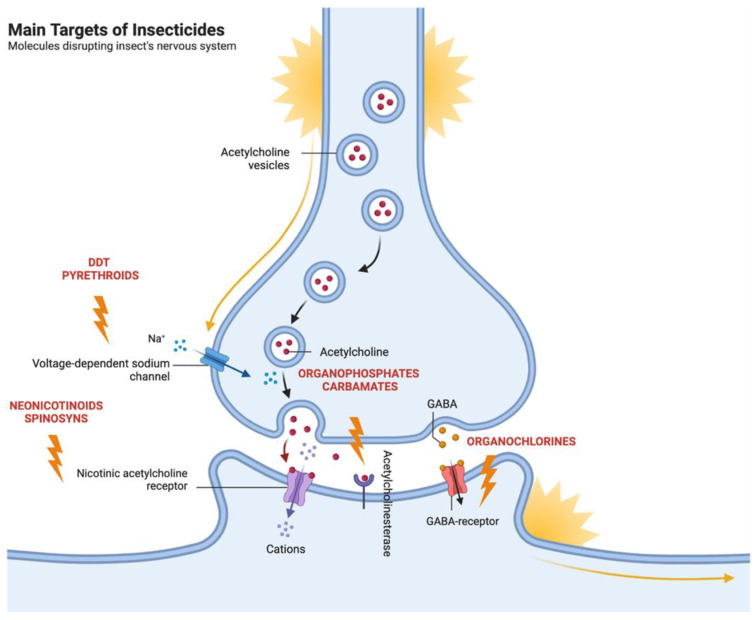
Schematic representation of the main targets and classes of insecticides that disrupt the insect nervous system. The diagram illustrates the common molecular targets, including (**a**) voltage-dependent sodium channels, (**b**) acetylcholinesterase, (**c**) GABA receptors, and (**d**) nicotinic acetylcholine receptors. The main classes of insecticides that act on these targets are also represented and include pyrethroids, DDT, organophosphates, carbamates, organochlorines, and neonicotinoids. These insecticides cause paralysis and the death of the insect by disrupting its nervous system.

Figure 1 illustrates the main targets and classes of insecticides that disrupt the insect nervous system.

### 4.2. Metabolic Targets

Other substances used as insecticides work as endotoxins and as highly toxic molecules; they interfere with the normal function of insect’s metabolisms. For instance, organosulfur compounds act as ovicides, eliminating the pest in the egg stage. They usually carry low toxicity to other organisms [63]. 

Dinitrophenols act by uncoupling or inhibiting oxidative phosphorylation, preventing the creation of the essential adenosine triphosphate (ATP) [64]. 

Organotins work similarly to dinitrophenol, attacking and inhibiting the same binding sites, preventing ATP formation. They are extensively used against mites on fruit trees, and they were formerly used as an antifouling agent and molluscicide, being highly toxic to aquatic life [65]. 

Pyrazoles act by inhibiting the NADH-CoQ reductase site of mitochondrial electron transport, which disrupts ATP formation [66].

Pyridazinones interrupt mitochondrial electron transport at site one and are mainly used as a miticide. However, like most others, they showcase toxicity to aquatic arthropods and fish [67,68]. 

Botanical preparations can also work as endotoxins and, depending on the type, can have various effects. Rotenone is a naturally occurring compound found in the roots, stems, and leaves of certain plants, including the *Derris* and *Lonchocarpus* species; acts as a respiratory enzyme inhibitor, and is used as a piscicide that kills fish at doses that are non-toxic to fish food organisms [69]. Neem is a tree that is native to India. The leaves, bark and seeds of the neem tree contain compounds with medicinal properties that are used as insecticides since they reduce feeding and disrupt moulting by inhibiting biosynthesis or metabolism of ecdysone, the moulting hormone [70]. It is commonly used against moth and butterfly larvae.

Fumigants act by releasing gas into the air, which penetrates the treated space and targets the metabolism of pests. These agrochemicals’ effect on metabolism can vary depending on the specific chemical and the target organism. However, in general, fumigants disrupt key metabolic pathways in target organisms, such as energy production, DNA synthesis, and protein synthesis, leading to cell death. Additionally, some fumigants can react with cellular components, such as proteins and DNA, causing structural damage that impairs metabolic function. Methyl bromide, phosphine, sulfuryl fluoride, and ethylene oxide constitute some commonly used fumigants [71].

Inorganic compounds can also be used as insecticides. Their mode of action is dependent on the type of inorganic compound. Typical examples include uncoupling oxidative phosphorylation (arsenicals), inhibition of enzymes involved in energy production, and acting as desiccants. For each pest group, there is a different compound to be applied according to its efficacy; for example, for mites, sulfur should be used, and for cockroaches, boric acid [72].

### 4.3. Growth Regulators and Others

Biochemicals, which are classified as biorational compounds, have low toxicity to non-targeted species [73] and consist of various substances such as hormones; enzymes; pheromones; growth regulators; and microbials such as viruses, bacteria, fungi, protozoa, and nematodes. They act as either attractants, growth regulators, or endotoxins and can also function as attractants to specific species [74,75]. As an example, benzoylureas act as insect growth regulators by interfering with chitin synthesis [76]. 

Quinazolines have been shown to affect the larval stages of many insects by hindering the production of chitin in the exoskeleton, which can lead to the breaking of the cuticle or death due to lack of food in the affected larvae [77,78]. 

There is also another set of compounds classified as synergists/activators, which inhibit cytochrome P450-dependent polysubstrate monooxygenases (PSMOs), preventing the degradation of toxicants and enhancing the activity of insecticides when used alongside them; synergists and activators are not themselves considered toxic or insecticidal. Examples include piperonyl butoxide (PBO) and *N*-octyl bicycloheptene dicarboximide (MGK-264) [79,80,81]. 

**Figure 2:**
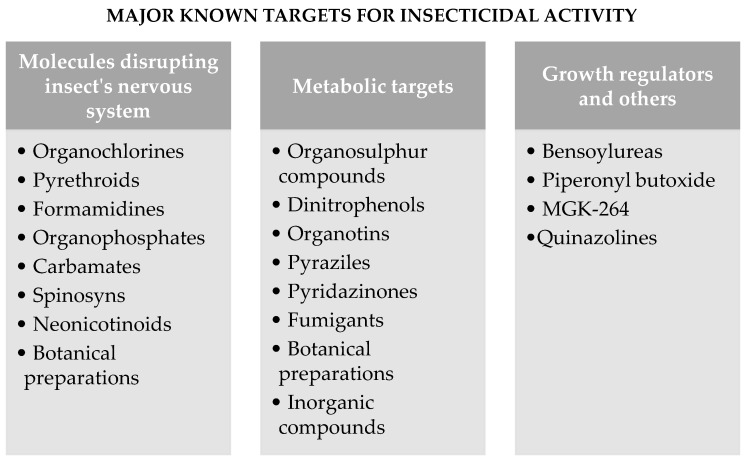
Major known targets for insecticidal activity.

Figure 2 shows the major known targets for insecticidal activity.

## 5. The Problem of Resistance

As a serious threat to human health and agriculture, insect pests can be controlled using insecticides; however, during this ongoing war, insects evolved and found ways to retaliate through the development of multiple resistances. Insects have short life cycles and produce numerous offspring, enabling them to adapt rapidly to stressful situations, such as exposure to insecticides. Their adaptability is due to their high potential for genetic variation, allowing for the evolution of traits that promote resistance. When an insect pest that is infesting a cultivation becomes resistant, farmers tend to increase the insecticide’s usage in quantity and on a larger scale. However, they must be replaced by other types of insect control as soon as possible, right when pest control diminishes [81]. The development of insect resistance can be attributed to three primary factors: genetics, biological and ecological factors, and operational practices. Genetics encompasses various elements, such as the frequency, number, and dominance of resistance alleles; penetrance, expressivity, and interactions of resistance alleles; past genetic selection through exposure to other chemicals; and the extent of integration of a resistant genome with fitness factors. Biological and ecological factors include biotic and generation turnover, offspring per generation, monogamy or polygamy, parthenogenesis, behavioral, isolation, mobility, migration, monophagy or polyphagy, fortuitous survival, and refugia. Lastly, operational practices encompass factors such as the chemical nature of the pesticide, interaction with previously used chemicals, persistence of residues, formulations, application threshold, selection threshold, life stages targeted, mode of application, space-limited selection, and alternating selection [82,83,84,85,86].

Typically, when an insect is exposed to an insecticide, the compound can rapidly penetrate the insect’s integument through various routes, such as contact, inhalation, or ingestion [87], ultimately reaching the intended target area. This could be a vital enzyme, nerve tissue, or receptor protein. After binding successfully, and when it reaches certain threshold concentrations, they cause a wide variety of symptoms, resulting in the insect’s death [88]. Resistance can be acquired at any step of the insecticide’s pathway. Thus, the rate of absorption could be lowered through acquiring higher levels of impermeability, while modifications could appear on target sites where the insecticide’s molecules no longer bind. In addition, the appearance of new or more enzymes to help break down the toxic compound is another way organisms can adapt to insecticides. When an organism is exposed to an insecticide, it may initially have low levels of the enzymes needed to break down the compound. However, over time, the organism may adapt and produce more of these enzymes, which can help to reduce the toxicity of the insecticide. This adaptation can be seen as an evolutionary response to the selective pressure of the insecticide, and it can lead to the emergence of resistant populations [89]. There is also a phenomenon called cross-resistance, where, when an arthropod develops a certain degree of resistance in relation to a compound, it is most likely that the same individual would be resistant to similarly acting insecticides [90]. For instance, there is the possibility that previous selection with insecticides can confer resistance to relatively new insecticides through cross-resistance [91]. One example of this is the diamondback moth, a common pest of cabbage and other cruciferous vegetables. Diamondback moths have become resistant to many insecticides over time, due in part to the extensive use of insecticides in agriculture. In one study, diamondback moths that had been exposed to pyrethroid insecticides were found to be cross-resistant to the newer neonicotinoid insecticides, even though the neonicotinoids had not been used extensively in the area. This suggests that the use of pyrethroids had selected for moths that were already resistant to neonicotinoids or that the two types of insecticides share similar mechanisms of action that contribute to cross-resistance [82].

Target-site resistance occurs when a specific insecticide’s site of action is modified within resistant insects, resulting in the molecules no longer binding effectively to those same sites [92]. This is often seen in important pest species with mutations at the nicotinic acetylcholine receptor (nAChR), which can lead to insensitivity to neonicotinoids [93]. Another form of resistance is altered target-site resistance, which happens when the site where the toxin usually binds becomes modified to reduce the insecticide’s effects. Insects achieve resistance through the modification of the target protein, resulting in a reduction in the binding of the insecticide and decreased effectiveness of the compound [94,95].

Metabolic resistance is the most common type of resistance and is characterized by a large set of enzymes that are used to breakdown the insecticide, normally used as a way for the insect to detoxify foreign materials. Resistant strains may possess higher levels or more effective forms of these enzymes [92]. 

The three main enzyme systems are esterases, mono-oxygenases, and glutathione S-transferases, and while metabolic resistance is important for all four insecticide classes (organophosphates, carbamates, pyrethroids, and neonicotinoids), different enzymes affect each class differently [96]. 

For example, multiple cytochrome P450s, which can structurally metabolize diverse substrates, are also known to play an important role in several biosynthetic pathways. These enzymes are involved in xenobiotic (insecticides and plant toxins) detoxification, hence promoting the development of insecticide resistance and insects’ adaptation to their hostplants [97]. 

Another type of resistance is behavioral [98]. As simple as it may seem, there are various reports of insects who stop feeding when they detect certain insecticides, even leaving the area where the toxic compound is abundant [99,100]. The capability of some insects to recognize danger and act accordingly is notable. Some may move to the underside of a sprayed leaf, move deeper into the crop’s canopy, or fly away from the contaminated area [100,101,102]. 

Cuticular resistance is traduced by the permeability level of the insect’s integument in relation to the toxic compound. The cuticle is the first and major barrier that protects the insect from penetration of external compounds. A reduced penetration of toxic compounds culminates in a reduced uptake of noxious chemicals; hence, modifications in the insect’s cuticle (such as in thickness) prevent or slow the absorption/penetration of insecticides [103]. 

Lastly, there is another physical resistance mechanism whereby the rate of excretion is increased. The excretion process can occur via reflex vomiting of the insecticide and/or defecation of the insecticide, with or without entry into the hemocoel. If the toxic compound is not able to enter hemocoel, the insecticide passes directly through the gut being excreted during defecation; if it does enter, however, the insecticide must be moved back into the lumen of the gut via filtration and efflux by the Malpighian tubules [104]. 

For circumventing the described resistance problems, it is highly advised to employ a synergistic approach combining alternative options for conventional frequently used insecticides.

## 6. Alternatives for Conventional Insecticides

Prolonged usage of synthetic insecticides has caused environmental damage, health problems, and biodiversity problems (such as loss of species diversity). Synthetic pesticides have also harmed farmers in the export trade, especially in the horticultural sector. Both farmers and exporters in developing countries have lost market and profits if banned insecticides were detected above the established tolerable level by law [83]. 

Some of the most rustic and/or outdated solutions for this expanding problem include cultural practices such as the implementation of physical or mechanical barriers (e.g., aluminum foil mulches, see-through nets, temperature/relative humidity manipulation, physical shock and electric discharges, etc.), crop rotations and intercropping (increasing crop diversity), alternative seeding patterns, and companion planting (companion plants which improve crop’s performance or work as a lure/repellent for insect pests) [84,85,86].

Genetically modified plants have long been considered a potential solution to the challenge of feeding a growing population while minimizing the negative impacts on the environment. One promising approach has been to develop plants with intrinsic insect resistance, which can help to reduce the use of chemical pesticides and ultimately decrease carbon dioxide emissions. To achieve this goal, researchers in plant resistance need to utilize advanced technologies such as genotyping by sequencing and high-throughput phenotyping. These tools allow for the identification, mapping, and tracking of important resistance genes in plants, which are essential for the development of new, improved crop varieties [87].

Additionally, besides engineered pest-resistant crops, genetic pest management includes genetic control of the pest itself. This is focused on sterility resulting from hybrid crosses between different species or different genetic strains that result from the action of mutagenic ionizing radiation. Presumably, this might be used to induce dominant lethal mutations in insects, which, when released into the wild, could sterilize female insects [88,89].

Microbial control is another promising method that involves using insect pathogens to manage pest populations. This approach identifies and utilizes host-virus associations, including various microbial agents, such as fungi, protozoa, bacteria, and nematodes. However, only a few of these entomopathogens have been developed into effective biocontrol agents, and developing new microbial agents requires rigorous testing to determine their efficacy and safety [90,91].

Nowadays, most studies focus on the exploitation of natural products since they offer several advantages over synthetic insecticides. One of the advantages of botanical insecticides is their efficacy against a broad range of insect pests. This is due to their diverse modes of action, which can include disrupting the insect’s nervous system, interfering with its metabolism, or causing physical damage to its outer shell. Another advantage is their biodegradability, which means they break down naturally and do not persist in the environment.

Botanical insecticides are also known for their low toxicity to non-target organisms, including humans, pets, and beneficial insects such as bees and butterflies. This makes them a safer option for pest control in areas where non-target organisms may be present [68].

One example of a botanical insecticide is eugenol, which is derived from clove oil. Eugenol has shown efficacy against a variety of insect pests, including aphids, mites, and whiteflies. It works by disrupting the insect’s nervous system, causing paralysis and death. Other examples of botanical insecticides include pyrethrins, which are derived from chrysanthemum flowers and are effective against a range of insects such as mosquitoes, flies, and moths. Another example is rotenone, which is extracted from the roots of several tropical plants and has been used for insect control for centuries. Table 2 compiles the biochemical sites of action of the most prominent natural insecticides [105].

In addition to their efficacy and safety, botanical insecticides offer a high accessibility of source materials, as many of the plants from which they are derived can be easily grown and harvested. This makes them a sustainable option for pest control, particularly in developing countries, where access to synthetic insecticides may be limited [105].

## 7. Conclusions

The development of insecticides has played a critical role in modern agriculture by providing effective control of pests, thereby ensuring food security and improving crop yields. As the world’s population continues to grow, it is imperative that agriculture remains productive and sustainable. However, the increasing resistance of pests to existing insecticides, as well as concerns over their environmental impact, highlights the need for continued research and innovation in this field.

To meet this challenge, future insecticide development is likely to focus on several key areas, including eco-friendly alternatives such as biopesticides and insect growth regulators, effective resistance management strategies, precision agriculture technologies that minimize the use of insecticides, combination products that target multiple modes of action, and the discovery of novel modes of action that will lead to the development of more effective and safer insecticides.

By addressing these areas, we can ensure that the future of insecticide development will not only maintain effective pest control but also promote sustainable agriculture and minimize negative impacts on the environment. Ultimately, this will help secure the long-term productivity of agriculture and the well-being of our planet and its inhabitants.

## Figures and Tables

**Table 1:** Structural and chemical composition, as well as corresponding adverse effects, of the ten currently most used insecticides in world.

Insecticide	Chemical Formula	Chemical Structure	Adverse Effects
(1) Imidacloprid (Neonicotinoid)	C_9_H_10_C_l_N_5_O_2_	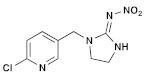	The residues of this substance can make their way into the food chain and affect both the reproductive capacity of lab rats and that of their offspring. It is a chemical that disrupts endocrine and steroidogenesis [33].
(2) Chlorpyrifos (Organophosphate)	C_9_H_11_C_l3_NO_3_PS	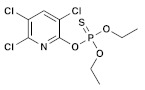	The laboratory rats that were exposed showed a decrease in body weight and an increase in the relative weights of their liver and kidney. The damage to their liver was significant, and there was a notable increase in total protein and uric acid levels. Additionally, there was an increase in oxidative stress observed in the exposed rats [34,35].
(3) Carbaryl (Carbamate)	C_12_H_11_NO_2_	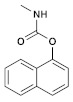	The toxicity observed is a result of cholinesterase inhibition. When pigs were exposed to this substance for an extended period, it caused a progressive neuromyopathy that resulted in structural damage, which cannot be reversed acutely with atropine. Similarly, in lab rats, there was a significant reduction in their overall weight. Moreover, there was a notable decrease in the number of germ cells, spermatocytes, spermatids, and Leydig cells. Additionally, the testosterone levels significantly declined, while the levels of LH and FSH increased significantly [36,37].
(4) Acephate (Organophosphate)	C_4_H_10_NO_3_PS	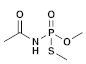	The highest doses administered to lab rats inhibited the activity of acetylcholinesterase in the brain and skeletal muscles. In the same group, there was a decrease in the number of implantations and live fetuses, along with an increase in the number of early resorptions observed. Furthermore, there was a decrease in sperm motility and count in the exposed rats. Dose-dependent histologic changes, including the degeneration of muscle fibers, were also observed [38].
(5) Dimethoate (Organophosphate)	C_5_H_12_NO_3_PS_2_	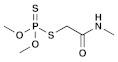	This substance, like other organophosphates, is known to inhibit acetylcholinesterase (AChE) activity, leading to severe nerve damage. In plants, its effects are reflected in reduced photosynthesis and growth, while in birds, the activity of brain enzymes is inhibited, resulting in sublethal effects. Aquatic organisms are expected to be highly affected by direct exposure, leading to changes in their swimming behavior [39].
(6) Thiamethoxam (Neonicotinoid)	C_8_H_10_ClN_5_O_3_S	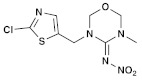 (*E* isomer) 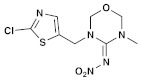 (*Z* isomer)	In cockerels, exposure to thiamethoxam (TMX) at sub-lethal levels resulted in a dose-dependent reduction in key hematological parameters, including total erythrocyte count, hemoglobin, packed cell volume, and total leukocyte count. The biochemistry of the birds was also impacted, with significant alterations in total proteins, albumin, and globulin. The study indicated that TMX caused substantial changes in the hematological profile and liver and kidney function of the birds. In addition, TMX increased oxidative damage to lipids and DNA in these organs, while reducing the antioxidant activities in liver and kidney cells, leading to oxidative stress [40,41].
(7) Malathion (Organophosphate)	C_10_H_19_O_6_PS_2_	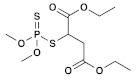	Malathion (MAL) was found to have adverse effects on frog oocyte maturation, resulting in reduced levels of Emi2, a critical factor for oocyte maturation. In addition, embryos fertilized under the influence of MAL showed a higher rate of abnormal division, leading to embryo death during early embryogenesis. The toxicity mechanisms of MAL include inhibition of acetylcholinesterase, oxidative stress, DNA damage, and apoptotic cell damage. Its toxic effects on the central nervous system are well documented, but it also affects the liver, kidney, testis, ovaries, lung, pancreas, and blood. MAL is considered a genotoxic and carcinogenic chemical compound and evidence shows adverse effects associated with prenatal, and postnatal exposure in both animals and humans. These findings are supported by various studies [42,43].
(8) Zeta-cypermethrin (Pyrethroid)	C_22_H_19_Cl_2_NO_3_	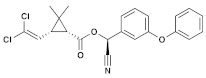	In common guppies (*Leporinus reticulatus*), exposure to various doses of zeta-cypermethrin resulted in the lifting of the epithelial layer from gill lamellae and necrosis. Other observed histopathological effects included exudation, hyperplasia, and shortening of secondary lamellae. Additionally, in vitro experiments showed that zeta-cypermethrin caused DNA damage in human peripheral lymphocytes, indicating its genotoxic properties [44,45].
(9) Bifenthrin (Pyrethroid)	C_23_H_22_ClF_3_O_2_	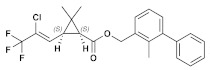	The hepatic function of tadpoles is negatively affected by cis-bifenthrin. Aquatic species are highly susceptible to the acute lethal toxicity of bifenthrin. Bifenthrin also has sublethal toxic effects on non-target organisms, such as developmental toxicity, neurobehavioral toxicity, oxidative damage, immune toxicity, and endocrine-disrupting effects [46,47].
(10) λ-cyhalothrin (Pyrethroid)	C_23_H_19_ClF_3_NO_3_	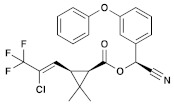	Previously conducted research has indicated that synthetic pyrethroids, such as λ-cyhalothrin (LCT), have high levels of aquatic toxicity. Exposure of zebrafish to synthetic pyrethroids, including LCT, resulted in a dose-dependent increase in mortality, higher malformation rates, and lower hatching rates. This exposure to LCT led to a significant decrease in thyroid hormone triiodothyronine (T3) levels, indicating potential developmental toxicity by disrupting endocrine signaling at concentrations present in the environment. In other studies, administration of LCT to laboratory rats led to decreased functional sperm parameters, enzymatic and non-enzymatic antioxidant levels, and the presence of irregular seminiferous tubules containing only Sertoli cells [48,49].

**Table 2:** Biochemical sites of action of natural insecticides [105].

Common Name	Class of Insecticide	Targeted System	Mode of Action
Abamectin	Avermectin	Nervous system	Chloride channel activator
Azadirachtin	Botanical from neem oil	Growth and development/metabolic processes	Prothoracicotropic hormone (PTTH) inhibitor; phagostimulant disruptor
*Bacillus thuringiensis*	Microbial	Metabolic processes	Insect midgut membrane disruptor
Cinnamaldehyde	Botanical	Energy production	Exact mode of action not well understood; possibly interference with glucose uptake or utilization
Decalesides I and II	Botanical (natural trisaccharides)	Nervous system	Inhibition of sodium pump
Emamectin benzoate	Avermectin	GABA-gated chloride channels	Chloride channel activators
Pyrethrins I and II	Botanical (pyrethrum)	Nervous system	Sodium channel modulator
Rotenone	Botanical	Mitochondrial electron transport system	Electron transport inhibitor—site 1
Ryanodine	Botanical	Calcium channels (ryanodine receptor)	Activation
Spinosad	Spinosyn	Nervous system	Nicotinic acetylcholine receptor agonist (mimic)

## Data Availability

Not applicable.
